# Altered Sleep-Related Consolidation and Neurocognitive Comorbidity in CECTS

**DOI:** 10.3389/fnhum.2021.563807

**Published:** 2021-06-07

**Authors:** Victoria Georgopoulou, Karen Spruyt, Kyriakos Garganis, Mary H. Kosmidis

**Affiliations:** ^1^2nd Centre for Educational and Counseling Support of Eastern Thessaloniki, Ministry of Education, Thessaloniki, Greece; ^2^Department of Educational and Social Policy, University of Macedonia, Thessaloniki, Greece; ^3^INSERM, Claude Bernard University, School of Medicine, Lyon, France; ^4^Epilepsy Unit, St Luke’s Hospital, Thessaloniki, Greece; ^5^Lab of Cognitive Neuroscience, School of Psychology, Aristotle University of Thessaloniki, Thessaloniki, Greece

**Keywords:** neuroplasticity, active system consolidation, neuronal oscillations, sleep electrophysiology, memory

## Abstract

Our aim is to use neurophysiological sleep-related consolidation (SRC) phenomena to identify putative pathophysiological mechanisms in CECTS linked to diffuse neurocognitive deficits. We argue that there are numerous studies on the association between seizure aspects and neurocognitive functioning but not as many on interictal variables and neurocognitive deficits. We suggest two additional foci. First, the interictal presentation in CECTS and second, neuronal oscillations involved in SRC processes. Existing data on mechanisms through which interictal epileptiform spikes (IES) impact upon SRC indicate that they have the potential to: (a) perturb cross-regional coupling of neuronal oscillations, (b) mimic consolidation processes, (c) alter the precision of the spatiotemporal coupling of oscillations, and (d) variably impact upon SRC performance. Sleep spindles merit systematic study in CECTS in order to clarify: (a) the state of the slow oscillations (SOs) with which they coordinate, (b) the precision of slow oscillation-spindle coupling, and (c) whether their developmental trajectories differ from those of healthy children. We subsequently review studies on the associations between IES load during NREM sleep and SRC performance in childhood epilepsy. We then use sleep consolidation neurophysiological processes and their interplay with IES to help clarify the diffuse neurocognitive deficits that have been empirically documented in CECTS. We claim that studying SRC in CECTS will help to clarify pathophysiological mechanisms toward diverse neurocognitive deficits. Future developments could include close links between the fields of epilepsy and sleep, as well as new therapeutic neurostimulation targets. At the clinical level, children diagnosed with CECTS could benefit from close monitoring with respect to epilepsy, sleep and neurocognitive functions.

## Introduction

### Childhood Epilepsy With Centrotemporal Spikes

Childhood epilepsy with centrotemporal spikes (CECTS) belongs to a common group of idiopathic focal childhood epilepsies (IFEs), often of genetic origin (Panjwani et al., 2016; Strug and Pal, 2017). It is conceptualized as an age-related neurodevelopmental dysfunction of a self-remitting nature (Miano and Datta, 2019). Traditionally, CECTS was thought to be free of structural abnormalities and this is still reflected in some of the diagnostic practices (Pavlou et al., 2012). However, evidence of subtle functional and structural abnormalities in CECTS has been mounting (Pardoe et al., 2013; Dryźałowski et al., 2018). Despite a growing consensus that neurocognitive impairment in CECTS subsides beyond the active phase (Deonna et al., 2000; Varesio et al., 2020), there is also evidence about verbal deficits in the remission period associated with persistent EEG abnormalities (Massa et al., 2001; Filippini et al., 2013). The onset of CECTS is between early childhood and middle adolescence. The incidence of the disorder is between 10 and 20: 100,000 in children between the ages of 3 and 15 (Parakh and Katewa, 2015; Smith et al., 2016) and its prevalence is approximately 15% in children with epilepsy between 1 and 15 years of age (Parakh and Katewa, 2015).

Centrotemporal spikes are a defining feature of CECTS and are documented through sleep-electroencephalogram (EEG) during NREM sleep (Pavlou et al., 2012). The negative impact of interictal epileptiform spikes (IES) on children’s neurocognitive profile has been substantiated through: (a) the increased prevalence of IES in children with developmental language (Neuschlová et al., 2007) or behavior disorders (Silvestri et al., 2007) and, (b) the contribution of IES to neurocognitive deficits in epileptic children when accounting for confounding variables (Fastenau et al., 2009). The evolution of the child’s neurocognitive profile partially depends on the spike index and the localization of IES (Nicolai et al., 2007; Zhao et al., 2007). Moreover, the frequency of IES is among the factors that determine whether CECTS will follow a typical or atypical trajectory (Kanemura et al., 2012).

### Sleep-Related Consolidation Processes

The synaptic homeostasis hypothesis (Tononi and Cirelli, 2006, 2014) purports that daytime tasks result in high synaptic potentiation in cortical neuronal networks. Slow wave activity (SWA) and slow oscillations (SOs) in particular, subsequently globally depotentiate the synapses that were potentiated. SWA hence has been reported to directly reflect the level of synaptic potentiation that took place during the previous waking period. This has stimulated several sleep-related consolidation (SRC) research protocols.

In the active system consolidation model (Rasch and Born, 2013) new stimuli are recorded simultaneously in two different “stores:” (a) a hippocampal fast-learning temporary “store” and, (b) a neocortical slow-learning long-term “store” (Rasch and Born, 2013). Through a process termed system consolidation, memory traces undergo significant qualitative transformations, as they move from hippocampal temporary neuronal networks into neocortical long term “storage” (Rasch and Born, 2008). Once they reach the neocortex, the newly reorganized memory representations need to be strengthened. A bulk of research has shown that Non-Rapid Eye Movement sleep (NREM), particularly slow wave sleep (SWS), plays a key role in enhancing declarative memory, which is hippocampus dependent. Sleep spindles are a characteristic feature of NREM sleep and have a “waxing and waning” electrophysiological representation (Weiner and Dang-Vu, 2016). An advantage of this model over others, is that it describes the electrophysiological and neurochemical mechanisms through which sleep enhances performance. These two models of SRC are not mutually exclusive.

Sleep-related consolidation, a type of neuroplasticity, correlates with cortical maturation and cognition (Kopasz et al., 2010). It has been studied in children with sleep disorders (Cellini, 2017), in children with attention deficit hyperactivity disorder (ADHD) (Prehn-Kristensen et al., 2011), and in children with epilepsy (Sud et al., 2014) including children with idiopathic focal epilepsies (Urbain et al., 2011; Galer et al., 2015).

### CECTS and Sleep-Related Consolidation

Studying SRC in CECTS is particularly relevant for a number of reasons. First, there is increasing consensus that CECTS and electrical status epilepticus during slow wave sleep (ESES), an IFE complicated by continuous spike and wave during sleep (CSWS), are within the same continuum, with ESES comprising the severe end of the continuum (Galer et al., 2015; Halász et al., 2019). It has been recognized that frequent and diffuse IES, such as those present in ESES, contribute to severe behavioral and cognitive deficits. The mechanism through which IES produce this result in ESES is potentially through SRC phenomena (Tassinari et al., 2009). CSWS EEG patterns are known to exist in CECTS, too, especially in atypical forms of the disorder (Parisi et al., 2017). Second, in CECTS, IES present mainly during NREM sleep; hence a time frame that is crucial for SRC processes, particularly in declarative memory.

It has been suggested that IES may interfere with the neurophysiological consolidation processes subserving learning. This hypothesis is strengthened by associations between behavioral and cognitive deficits and IES severity at the acute epileptic phase (Storz et al., 2020). It is also supported by a pilot study documenting that an IES-free EEG signal tends to revert the cognitive deficits (Urbain et al., 2011). Furthermore, the lateralization and localization of IES are among the factors that impact upon SRC in children with IFE (Galer et al., 2015). Therefore, CECTS can serve as a model to elucidate whether, due to epilepsy-related phenomena and alterations in neuronal networks, there is a subsequent alteration in the SRC processes leading to neurocognitive deficits.

## Ies and Sleep-Related Consolidation Performance in Childhood Epilepsy

Associations between IES load during NREM sleep and SRC performance in various types of childhood epilepsy have been found in a limited number of studies so far (Table 1). Collectively these studies advance the field by (a) differentiating the impact of sleep from that of a wake delay, (b) delineating associations between IES load and sleep-related gains in childhood epilepsy, (c) demonstrating that home measurements are producible in the unfamiliar hospital setting, and (d) by bridging SRC performance data in idiopathic and structural childhood epilepsies. In relation to the latter point, an inverse correlation between IES load and SRC in structural childhood epilepsy was suggested as a pathophysiological mechanism bridging findings from studies of structural and idiopathic focal epilepsies (Chan et al., 2017). The most common limitations in this type of study are heterogeneous sampling, small sample size, pharmacotherapy variations and occasionally the lack of a control group. Future studies could add electrophysiological data and in particular data reflecting the function of the three cardinal neuronal oscillations in SRC processes.

**TABLE 1:** Studies on IEDs and SRC performance in childhood epilepsy.

**Authors, year**	**Type of childhood epilepsy**	**MRI**	**Sample size (n)**	**Age**	**Gender (male)**	**Memory assessment**	**Experimental design**	**Overnight recordings**
Galer et al. (2015)	IFE	No structural abnormality	CE = 15; Control = 8;	CE = 6–12; Control = 7–12	CE = 9; Control = 3	V = 6; NV = 5; V + NV = 4	Immediate retrieval vs. to post sleep retrieval; home condition vs. hospital condition	Polysomnography; EEG
	**Info on IEDs:** The frequency of IEDs during NREM sleep was computed by calculating a spike-wave index; IED quantification on the first 30 min of NREM sleep was reliable to assess the rate of interictal spiking during NREM sleep							
	**Conclusion CE:** ↓ V performance and NV performance; A significantly positive association between the SWI and the extent of the deficit in the NV performance; home performance = hospital performance							
Chan et al. (2017)	Focal pharmaco-resistant epilepsy with structural etiology	Structural abnormalities	CE = 22; Control = 21	CE = 6–16; Control = 6–16	CE = 13; Control = 9	V and NV	A within-subject comparison of memory retention across intervals of wake or overnight sleep; average interval between learning and testing was 15 h	EEG; polysomnography
	**Info on IEDs:** IEDs included sharp waves, spikes, spike and slow wave complexes, and polyspike- and slow wave complexes; The count was recorded as the number of IEDs per minute, calculated by dividing the total number of IEDs by the duration of sleep time over which they were counted							
	**Conclusion CE:** in the sleep condition ↑ V and NV performance. V and NV performance negatively correlated with IEDs rate; a longer history of epilepsy associated with greater contribution of sleep to V performance							
	**Conclusion Control:** in the sleep condition ↑ V and NV performance							
Sud et al. (2014)	Refractory epilepsy	4 out of 10 with structural abnormalities	CE = 10	CE = 8–18	CE = 7	V	A within-subject comparison of memory retention across intervals of wake or overnight sleep; No control group	Continuous video-EEG; actigraphy
	**Info on IEDs:** A spike index was done in a blinded fashion by one of the researchers to determine the number of IEDs; Interictal spike frequency was visually for 25 min (5 × 5 epochs) randomly selected during the waking and NREM sleep periods; The average spike index was calculated (combining sleep and wake epochs) for spike/min							
	**Conclusion CE:** No ↑ in V performance after a period of sleep than after a wake period							
Urbain et al. (2011)	IFE	No structural abnormality	CE = 4 (only tested in the sleep condition); sleep control = 12; wake control = 12	CE = 7–10; control participants matched to patients for age	CE = 3; sleep control = 8; wake control = 7	V	Comparing the wake to the sleep condition in healthy controls; comparing the healthy sleep condition to the patient sleep condition	Video EEG
	**Info on IEDs:** Sleep EEG was analyzed qualitatively using a grading system and quantitatively in calculating SWI in stages 1 and 2 of NREM sleep							
	**Conclusion CE:**↓ V performance after sleep							
	**Conclusion Control:** ↑ V performance after sleep; ↓ V performance after wake interval							
Storz et al. (2020)	Self-limited focal epilepsy	Not reported	CE = 14; Control = 15 (Wilhelm et al., 2008)	CE = 5.5–11.6; Control = 6.8–9.1	CE = 10; Control = 6	V and NV	Comparing immediate performance to that after a night’s sleep; whether IEDs rate in NREM sleep predicts retention performance	EEG; EOG; EMG
	**Info on IEDs:** In patient EEGs, sleep stages were scored visually; IEDs were visually detected in a bipolar and an average referenced montage of the standard 10–20 electrodes by a pediatric epileptologist; A SWI was then calculated by dividing the number of seconds affected by spike waves by the number of seconds analyzed multiplied by 100							
	**Conclusion CE:**↓ V performance after a night’s sleep; Positive association between SWI rates and V performance deficit; No overnight NV performance change; SWI rates not correlated with NV performance							
	**Conclusion Control:** ↑ V performance after a night’s sleep; No VN performance overnight change of performance							

****IEDs** = interictal epileptiform discharges; **IFE** = idiopathic focal epilepsy; **CE** = childhood epilepsy; **V** = verbal declarative memory task (word-pair learning task); **NV** = non-verbal declarative memory tasl (2D object location task); **EEG** = electroencephalogram; EOG, electrooculogram; **EMG** = electromyogram; **SWI** = spike wave index; ↑, increased; ↓, decreased.**

## Putative Pathophysiological Mechanisms Based on Electrophysiological Phenomena

During NREM sleep, neuronal networks from different brain regions “converse” through the temporal coupling of their respective electrophysiological features (Buzsáki, 1996). Phase amplitude coupling (PAC) refers to the temporal precision with which one oscillation modulates its amplitude by the phase of another oscillation. There is now evidence that PAC is crucial in SRC; in old age and in certain pathological conditions, oscillations lose the ability to interact in a temporally precise way (Muehlroth et al., 2019). More specifically, temporal coupling involves thalamic sleep spindles, hippocampal ripples and neocortical slow oscillations (SOs) (Buzsáki, 1996). The temporal co-ordination of these key electrophysiological features involves two temporal frames: (a) a top-down, and (b) a bottom-up (McDevit et al., 2017). During the top-down frame, the neocortical SOs act as a “metronome;” during certain phases they suppress spindles and ripples, while during other phases there is a rebound in the expression of the latter (McDevit et al., 2017). The bottom-up frame is an intricate process, during which hippocampal ripples nest within succeeding troughs of spindles and thus, convey replayed memory traces to spindles (Staresina et al., 2015). Next, spindles “pass on” this information to the neocortex. We suggest that each one of them should be studied systematically in CECTS, and herewith advocate sleep studies in children with epilepsy.

### Interictal Epileptiform Spikes Issues

#### Perturbing (Cross-)Regional Coupling

Direct intracranial EEG recordings have provided evidence of the coupling of SOs, spindles and ripples within the human hippocampus during NREM sleep (Staresina et al., 2015). The aforementioned coupling was possible because SOs and spindles appear simultaneously across vertex electrode Cz on the EEG and the hippocampus (cross-regional coupling) (Staresina et al., 2015). IES in CECTS, due to their centrotemporal location and NREM occurrence, may perturb one or more of the oscillations within the hippocampal area as well as their neuronal coupling potentially leading to neurocognitive deficits. This is partially supported by the fact that in adults, hippocampal interictal spikes in NREM sleep negatively correlate with memory consolidation performance (Lambert et al., 2020).

#### Mimicking Consolidation Processes

IES undergo sleep-related reactivation and consolidation processes utilizing physiological mechanisms that were considered to be reserved for meaningful stimuli (Bower et al., 2017). To date, data are available only from patients with refractory epilepsy. The pathological patterns of the IES were found to be preferentially reactivated during SWS; hence strengthened through physiological consolidation mechanisms (Bower et al., 2017). The aforementioned findings offer a new perspective on how epilepsy interacts with sleep-related neuroplasticity mechanisms and highlight the importance of collecting IES data.

#### Altering the Precision of the Spatiotemporal Co-ordination

Based on adult studies IES couple with slow wave oscillations at different transitional points (up to down states) in comparison to what physiological oscillations do (down to up states) (Frauscher and Gotman, 2019). In healthy children and adolescents spindle power decreases during the “down state” and increases during the “up-state;” hence there is an influence of the SO phase on spindle activity across development (Hahn et al., 2020). Hence, SRC and IES data collection in CECTS is pertinent in order to investigate whether the aforementioned normative processes are followed in this clinical group.

#### Variably Impacting Upon SRC Performance

In epileptogenesis the impact of IES, ranging from proictal to antiictogenic, does not depend only on IES characteristics but also on the complex interaction between IES and specific neuronal networks (Chvojka et al., 2019). In relation to SRC processes, the complex interplay of IES with the three cardinal neuronal oscillations may partially explain (a) why it is difficult to find clear causal links between IES characteristics and alterations in SRC performance and (b) the diverse neurocognitive outcomes.

### Sleep Spindles Issues

Sleep spindles merit systematic study in CECTS. Namely, within the hierarchical nesting of one oscillation within the other, spindles play an intermediary role since they are organized by the SOs upstates (in adults) and in their turn they group ripples within their own troughs (McDevit et al., 2017). In children too, there is an influence of the SO phase on spindle activity (Hahn et al., 2020). It is possible that some pathological alterations in sleep spindles in CECTS may jeopardize the efficient transfer of information through the hierarchical nesting of oscillations.

One important line of research would be to concurrently study the characteristics of sleep spindles in CECTS, the state of SOs with which they co-ordinate and NREM sleep-related gains. In fact, sleep spindle abnormalities have already been found in various clinical populations such as in mental disability (Shibagaki et al., 1982), autistic spectrum disorder (ASD) (Limoges et al., 2005), sleep disorders (Bove et al., 1994) and mental illness (Wamsley et al., 2012). Given that in CECTS some of the pathological phenomena, such as IES, occur predominantly at night time, the need arises to systematically study sleep spindles within this clinical population.

### Neocortical SO Phase Amplitude Coupling Issues

Precise SO-spindle coupling, detected at scalp level, is crucial for consolidation: (a) frontal spindles are thought to be time-locked to SOs “upstates” during childhood and adolescence; (b) the precision of SO-spindle coupling increases in parallel with brain maturation, especially in fronto-parietal areas and (c) subjects whose coupling strength increased from childhood to adolescence also presented with improved SRC performance in adolescence (Hahn et al., 2020). In adulthood too, the spatiotemporal precision of SO-spindle coupling is essential in enabling the consolidation of declarative memories (Muehlroth et al., 2019). In CECTS the properties of SO-spindle precision may partially explain long term deficits.

## Discussion

CECTS is associated with a range of neurocognitive deficits and of varied severity. Deficits have been documented in executive functions (Filippini et al., 2016), attentional processes (Deltour et al., 2007), memory (Verrotti et al., 2014) language (Teixeira and Santos, 2018), reading comprehension (Currie et al., 2018) and neuropsychiatric comorbidities (Ross et al., 2020). A review of studies on neurocognitive functioning in CECTS reported lower outcomes in this clinical population compared to healthy subjects in a wide range of cognitive functions. Specifically, there was a large effect on long-term storage and retrieval, moderate effects on overall intelligence quotient (IQ), acquired knowledge, fluid reasoning, short-term memory, processing speed, and fluency in retrieval and a small effect in visual processing (Wickens et al., 2017). Of note, even general intelligence ability presented deficits, challenging the traditional view that in CECTS, the IQ remains within the normal range. This might be partially explained by the exclusion criteria of many studies in CECTS, excluding children with low general intellectual ability (Wickens et al., 2017). Alterations in synaptic homeostasis in CECTS may disrupt neuropsychological functioning by placing competing demands on SRC processes (Chan et al., 2021).

There are two commonly hypothesized reasons for the diffusely impaired neurocognitive profile in CECTS. The first one is that IES propagate; hence their effect expands beyond the area of initial presentation (Chvojka et al., 2019). The second reason is related to the myriad of concurrent developmental processes that take place in the young organism’s brain. To mention but a few, there are adaptive and maladaptive developmental processes in: (a) sleep regulation (Bathory and Tomopoulos, 2017), (b) ontogenetic trajectories of specific neuronal oscillations (Clawson et al., 2016), (c) cortical maturation (Selvitelli et al., 2009; Fujiwara et al., 2018; Siripornpanich et al., 2019), (d) cognitive maturation (Kolb, 2018), and (e) topographical evolution of NREM sleep phenomena moving along a posterior to anterior axis (Kurth et al., 2010). The onset of CECTS plays a key role as to which neuronal networks and which functions will be affected. For instance, functions that have reached a relatively mature level by the time CECTS commences might be less affected than functions that are at a crucial stage in their formation (Jurkevièiene et al., 2012). Given the sheer importance of developmental issues in the interplay of CECTS and SRC, it is noteworthy that many theories and datasets are adult-based. This research gap could be addressed by longitudinal prospective studies of CECTS that would simultaneously gather electrophysiological data on IES and neuronal oscillations, as well as behavioral data (sleep-related gains in learning material, particularly declarative).

From a neurophysiological perspective, deficits in general intelligence in CECTS could be tentatively explained by the fact that sleep spindles have been identified as an index of intelligence or general aptitude for learning (Fogel and Smith, 2011). A gap in the research is the relative lack of normative data on the developmental trajectories of sleep spindles with few notable exceptions (Scholle et al., 2007; Mcclain et al., 2016; Gorgoni et al., 2020). One approach would be to collect normative data on key features of sleep spindles across development. Another approach would be to consider whether sleep spindles have sufficient plasticity so as to adjust to their neurophysiological environment, namely the characteristics of the hippocampal ripples and the SOs, in order to optimize PAC and SRC.

In the latter case scenario, there are interesting implications for SRC processes in CECTS. Even within a neuronal environment burdened by IES, compensatory mechanisms could still enable sleep spindles to adjust their characteristics in order to achieve SRC processes. Hence, this would further support heterogeneous results in terms of SRC alterations in CECTS, possibly leading to heterogeneous long-term neurocognitive profiles.

Hippocampus-dependent memory is traditionally studied using declarative memory tasks including verbal and visuospatial tasks (non-verbal). Initial findings suggest that SRC is altered in CECTS (Urbain et al., 2011; Galer et al., 2015; Storz et al., 2020) meaning that language and visuospatial deficits could be partially explained on the basis of long-term problematic consolidation of this type of material. Indeed, a measure that involved IES in NREM sleep was associated with poorer performance on a visuospatial task in IFE subjects (Galer et al., 2015). Attentional deficits have been linked to altered NREM sleep in another clinical group, children with ADHD (Urbain et al., 2013). Attentional deficits in CECTS could thus be at least partially attributed to altered SRC processes. Language deficits are similarly expected in CECTS, especially when the centrotemporal IES are located in the left hemisphere, given that these brain areas are linked to language skills and processing of auditory stimuli (Teixeira and Santos, 2018; Halász et al., 2019). To date, the initial studies in SRC in CECTS in sleep-related gains were hampered by small numbers and sample heterogeneity. The field would benefit from replicating these studies with substantial samples and possibly with more than a single night’s sleep recording.

An additional challenge in CECTS is that since there is no clinical need for intracranial recordings, there is a lack of direct recordings of some types of neuronal oscillations. In order to overcome this issue well developed theoretical models are needed with testable hypothesis. There is a also a need to develop measures that can be detected at scalp level, such as the SO-spindle coupling measure based on which data can be inferred related to the quality of the hippocampal-neocortical communication (Helfrich et al., 2019). Future developments could include close links between the epilepsy and the sleep research fields. Sleep disruption is known to impact upon the neurophysiological mechanisms of SRC as well as upon EEG discharges (Parisi et al., 2017). At the same time, children with sleep-disordered breathing present an increased risk of neurocognitive deficits (Leng et al., 2017; Spruyt, 2021).

It is crucial that the contribution of each neuronal oscillatory pattern to the SRC process is well understood so that children with CECTS might be able to benefit from the fast developing field of sleep memory manipulation, and, in particular, of oscillatory sleep patterns. In adults various neurostimulatory methods, such as transcranial direct stimulation (Marshall et al., 2004) and acoustic stimulation (Ngo et al., 2013) boost SOs and spindles and concurrently increase sleep-related declarative memory gains (Marshall et al., 2006; Ngo et al., 2013). Future therapeutic approaches might include increasing SO or spindle intensity in CECTS in order to increase sleep-related gains in declarative memory tasks. Early stabilization of SRC processes might in turn avert long-term neurocognitive deficits leading to a better prognosis and quality of life for this vulnerable clinical group.

We propose that studying SRC in the context of CECTS will give us further insight into the pathophysiological mechanisms that lead to neurocognitive deficits in this pediatric population in the long term. At the clinical level, children diagnosed with CECTS could benefit from closely monitoring with respect to their epilepsy, sleep and neurocognitive functions.

## Author Contributions

VG conceived the work, searched and studied the literature, and drafted the first version of the manuscript. KS restructured, and discussed the literature and concepts with VG which resulted in the revised version. MK and KG commented on and reviewed the manuscript. All authors contributed to the article and approved the submitted version.

## Conflict of Interest

The authors declare that the research was conducted in the absence of any commercial or financial relationships that could be construed as a potential conflict of interest. The handling editor declared a past co-authorship with one of the authors KG.

## Abbreviations

CECTS: childhood epilepsy with centrotemporal spikes
IFE: idiopathic focal epilepsy
EEG: electroencephalogram
IES: interictal epileptiform spikes
SWA: slow wave activity
NREM sleep: non-rapid eye movement sleep
SWS: slow wave sleep
SRC: sleep-related consolidation
ADHD: attention deficit hyperactivity disorder
ESES: electrical status epilepticus during slow wave sleep
CSWS: continuous spike and wave during sleep
PAC: phase amplitude coupling
SOs: slow oscillations
ASD: autism spectrum disorder
IQ: intelligence quotient.

## References

[B1] Bathory E. Tomopoulos S. (2017). Sleep regulation, physiology and development, sleep duration and patterns, and sleep hygiene in infants, toddlers, and preschool-age children. *Curr. Probl. Pediatr. Adolesc. Health Care* 47 29–42. 10.1016/j.cppeds.2016.12.001 28117135

[B2] Bove A. Culebras A. Moore J. T. Westlake R. E. (1994). Relationship between sleep spindles and hypersomnia. *Sleep* 17 449–455. 10.1093/sleep/17.5.449 7991957

[B3] Bower M. R. Kucewicz M. T. St. Louis E. K. Meyer F. B. Marsh W. R. Stead M. (2017). Reactivation of seizure-related changes to interictal spike shape and synchrony during postseizure sleep in patients. *Epilepsia* 58 94–104. 10.1111/epi.13614 27859029 PMC5358812

[B4] Buzsáki G. (1996). The hippocampo-neocortical dialogue. *Cereb. Cortex* 6 81–92. 8670641 10.1093/cercor/6.2.81

[B5] Cellini N. (2017). Memory consolidation in sleep disorders. *Sleep Med. Rev.* 35 101–112. 10.1016/j.smrv.2016.09.003 27765468

[B6] Chan S. Chevalier C. Eriksson M. E. Baldeweg T. Cross J. H. (2021). “Sleep homeostasis, cognition and seizure propensity in children with focal epilepsy: insights from sleep deprivation,” in *Poster at the IPSA Congress 2021.Virtual.*

[B7] Chan S. Pressler R. Boyd S. G. Baldeweg T. Cross J. H. (2017). Does sleep benefit memory consolidation in children with focal epilepsy? *Epilepsia* 58 456–466. 10.1111/epi.13668 28111743

[B8] Chvojka J. Kudlacek J. Chang W. -C. Novak O. Tomaska F. Otahal J. (2019). The role of interictal discharges in ictogenesis – A dynamical perspective. *Epilepsy Behav.* 10.1016/j.yebeh.2019.106591 [Epub ahead of print]. 31806490

[B9] Clawson B. C. Durkin J. Aton S. J. (2016). Form and function of sleep spindles across the lifespan. *Neural Plast.* 2016:6936381. 10.1155/2016/6936381 27190654 PMC4848449

[B10] Currie N. K. Lew A. R. Palmer T. M. Basu H. De Goede C. Iyer A. (2018). Reading comprehension difficulties in children with rolandic epilepsy. *Dev. Med. Child Neurol.* 60 275–282. 10.1111/dmcn.13628 29238964

[B11] Deltour L. Quaglino V. Barathon M. De Broca A. Berquin P. (2007). Clinical evaluation of attentional processes in children with benign childhood epilepsy with centrotemporal spikes (BCECTS). *Epileptic Disord.* 9 424–431. 10.1684/epd.2007.0127 18077229

[B12] Deonna T. Zesiger P. Davidoff V. Maeder M. Mayor C. Roulet E. (2000). Benign partial epilepsy of childhood: a longitudinal neuropsychological and EEG study of cognitive function. *Dev. Med. Child Neurol.* 42 595–603. 10.1017/S0012162200001122 11034452

[B13] Dryźałowski P. Jóźwiak S. Franckiewicz M. Strzelecka J. (2018). Benign epilepsy with centrotemporal spikes – current concepts of diagnosis and treatment. *Neurol. Neurochir. Pol.* 52 677–689. 10.1016/j.pjnns.2018.08.010 30219586

[B14] Fastenau P. S. Johnson C. S. Perkins S. M. Byars A. W. DeGrauw T. J. Austin J. K. (2009). Neuropsychological status at seizure onset in children: risk factors for early cognitive deficits. *Neurology* 73 526–534. 10.1212/WNL.0b013e3181b23551 19675309 PMC2730794

[B15] Filippini M. Ardu E. Stefanelli S. Boni A. Gobbi G. Benso F. (2016). Neuropsychological profile in new-onset benign epilepsy with centrotemporal spikes (BECTS): focusing on executive functions. *Epilepsy Behav.* 54 71–79. 10.1016/j.yebeh.2015.11.010 26667848

[B16] Filippini M. Boni A. Giannotta M. Gobbi G. (2013). Neuropsychological development in children belonging to BECTS spectrum: long-term effect of epileptiform activity. *Epilepsy Behav.* 28 504–511. 10.1016/j.yebeh.2013.06.016 23896351

[B17] Fogel S. M. Smith C. T. (2011). The function of the sleep spindle: a physiological index of intelligence and a mechanism for sleep-dependent memory consolidation. *Neurosci. Biobehav. Rev.* 35 1154–1165. 10.1016/j.neubiorev.2010.12.003 21167865

[B18] Frauscher B. Gotman J. (2019). Sleep, oscillations, interictal discharges, and seizures in human focal epilepsy. *Neurobiol. Dis.* 127 545–553. 10.1016/j.nbd.2019.04.007 30981828

[B19] Fujiwara H. Tenney J. Kadis D. S. Byars A. Altaye M. Spencer C. (2018). Cortical morphology, epileptiform discharges, and neuropsychological performance in BECTS. *Acta Neurol. Scand.* 138 432–440. 10.1111/ane.12997 29989147 PMC6175642

[B20] Galer S. Urbain C. De Tiège X. Emeriau M. Leproult R. Deliens G. (2015). Impaired sleep-related consolidation of declarative memories in idiopathic focal epilepsies of childhood. *Epilepsy Behav.* 43 16–23. 10.1016/j.yebeh.2014.11.032 25546732

[B21] Gorgoni M. D’Atri A. Scarpelli S. Reda F. De Gennaro L. (2020). Sleep electroencephalography and brain maturation: developmental trajectories and the relation with cognitive functioning. *Sleep Med.* 66 33–50. 10.1016/j.sleep.2019.06.025 31786427

[B22] Hahn M. A. Heib D. Schabus M. Hoedlmoser K. Helfrich R. F. (2020). Slow oscillation-spindle coupling predicts enhanced memory formation from childhood to adolescence. *ELife* 9:e53730. 10.7554/eLife.53730 32579108 PMC7314542

[B23] Halász P. Bódizs R. Ujma P. P. Fabó D. Szûcs A. (2019). Strong relationship between NREM sleep, epilepsy and plastic functions – a conceptual review on the neurophysiology background. *Epilepsy Res.* 150 95–105. 10.1016/j.eplepsyres.2018.11.008 30712997

[B24] Helfrich R. F. Lendner J. D. Mander B. A. Guillen H. Paff M. Mnatsakanyan L. (2019). Bidirectional prefrontal-hippocampal dynamics organize information transfer during sleep in humans. *Nat. Commun.* 10:3572. 10.1038/s41467-019-11444-x 31395890 PMC6687745

[B25] Jurkevièiene G. Endziniene M. Laukiene I. Šaferis V. Rastenyte D. Plioplys S. (2012). Association of language dysfunction and age of onset of benign epilepsy with centrotemporal spikes in children. *Eur. J. Paediatr. Neurol.* 16 653–661. 10.1016/j.ejpn.2012.03.011 22560726

[B26] Kanemura H. Sano F. Aoyagi K. Sugita K. Aihara M. (2012). Do sequential EEG changes predict atypical clinical features in rolandic epilepsy? *Dev. Med. Child Neurol.* 54 912–917. 10.1111/j.1469-8749.2012.04358.x 22759211

[B27] Kolb B. (2018). “Brain plasticity and experience,” in *The Neurobiology of Brain and Behavioral Development*, eds Gibb R. Kolb B. (London: Academic Press), 341–389. 10.1016/B978-0-12-804036-2.00013-3

[B28] Kopasz M. Loessl B. Hornyak M. Riemann D. Nissen C. Piosczyk H. (2010). Sleep and memory in healthy children and adolescents – a critical review. *Sleep Med. Rev.* 14 167–177. 10.1016/j.smrv.2009.10.006 20093053

[B29] Kurth S. Ringli M. Geiger A. LeBourgeois M. Jenni O. G. Huber R. (2010). Mapping of cortical activity in the first two decades of life: a high-density sleep electroencephalogram study. *J. Neurosci.* 30 13211–13219. 10.1523/JNEUROSCI.2532-10.2010 20926647 PMC3010358

[B30] Lambert I. Tramoni-Negre E. Lagarde S. Roehri N. Giusiano B. Trebuchon-Da Fonseca A. (2020). Hippocampal interictal spikes during sleep impact long-term memory consolidation. *Ann. Neurol.* 87 976–987. 10.1002/ana.25744 32279329

[B31] Leng Y. McEvoy C. T. Allen I. E. Yaffe K. (2017). Association of sleep-disordered breathing with cognitive function and risk of cognitive impairment: a systematic review and meta-analysis. *JAMA Neurol.* 74 1237–1245. 10.1001/jamaneurol.2017.2180 28846764 PMC5710301

[B32] Limoges É Mottron L. Bolduc C. Berthiaume C. Godbout R. (2005). Atypical sleep architecture and the autism phenotype. *Brain* 128 1049–1061. 10.1093/brain/awh425 15705609

[B33] Marshall L. Helgadóttir H. Mölle M. Born J. (2006). Boosting slow oscillations during sleep potentiates memory. *Nature* 444 610–613. 10.1038/nature05278 17086200

[B34] Marshall L. Mölle M. Hallschmid M. Born J. (2004). Transcranial direct current stimulation during sleep improves declarative memory. *J. Neurosci.* 24 9985–9992. 10.1523/JNEUROSCI.2725-04.2004 15525784 PMC6730231

[B35] Massa R. De Saint-Martin A. Carcangiu R. Rudolf G. Seegmuller C. Kleitz C. (2001). EEG criteria predictive of complicated evolution in idiopathic rolandic epilepsy. *Neurology* 57 1071–1079. 10.1212/WNL.57.6.1071 11571336

[B36] Mcclain I. J. Lustenberger C. Achermann P. Lassonde J. M. Kurth S. Lebourgeois M. K. (2016). Developmental changes in sleep spindle characteristics and sigma power across early childhood. *Neural Plast.* 2016:3670951. 10.1155/2016/3670951 27110405 PMC4826705

[B37] McDevit E. A. Krishnan G. P. Bazhenov M. Mednick S. C. (2017). “The role of sleep spindles in sleep-dependent memory consolidation,” in *Cognitive Neuroscience of Memory Consolidation. Studies in Neuroscience, Psychology and Behavioural Economics*, Vol. 7 eds Axmacher N. Rasch B. (Switzerland: Springer International), 209–226. 10.1007/978-3-319-45066-7

[B38] Miano S. Datta A. N. (2019). The role of sleep-related cognitive functions in the spectrum of benign epilepsy with centro-temporal spikes. *Eur. J. Pediatr.* 178 1129–1137. 10.1007/s00431-019-03413-9 31227889

[B39] Muehlroth B. E. Sander M. C. Fandakova Y. Grandy T. H. Rasch B. Shing Y. L. (2019). Precise slow oscillation–spindle coupling promotes memory consolidation in younger and older adults. *Sci. Rep.* 9:1940. 10.1038/s41598-018-36557-z 30760741 PMC6374430

[B40] Neuschlová L. Štěrbová K. Žáčková J. Komárek V. (2007). Epileptiform activity in children with developmental dysphasia: quantification of discharges in overnight sleep video-EEG. *Epileptic Disord.* 9(Suppl. 1) S28–S35. 10.1684/epd.2007.0153 18319198

[B41] Ngo H. V. V. Claussen J. C. Born J. Mölle M. (2013). Induction of slow oscillations by rhythmic acoustic stimulation. *J. Sleep Res.* 22 22–31. 10.1111/j.1365-2869.2012.01039.x 22913273

[B42] Nicolai J. Van Der Linden I. Arends J. B. A. M. Van Mil S. G. M. Weber J. W. Vles J. S. H. (2007). EEG characteristics related to educational impairments in children with benign childhood epilepsy with centrotemporal spikes. *Epilepsia* 48 2093–2100. 10.1111/j.1528-1167.2007.01203.x 17645539

[B43] Panjwani N. Wilson M. D. Addis L. Crosbie J. Wirrell E. Auvin S. (2016). A microRNA-328 binding site in PAX6 is associated with centrotemporal spikes of rolandic epilepsy. *Ann. Clin. Transl. Neurol.* 3 512–522. 10.1002/acn3.320 27386500 PMC4931716

[B44] Parakh M. Katewa V. (2015). A review of the not so benign- benign childhood epilepsy with centrotemporal spikes. *J. Neurol. Neurophysiol.* 6 1–4. 10.4172/2155-9562.100031426753104

[B45] Pardoe H. R. Berg A. T. Archer J. S. Fulbright R. K. Jackson G. D. (2013). A neurodevelopmental basis for BECTS: evidence from structural MRI. *Epilepsy Res.* 105 133–139. 10.1016/j.eplepsyres.2012.11.008 23375559 PMC3669634

[B46] Parisi P. Paolino M. C. Raucci U. Ferretti A. Villa M. P. Trenite D. K. N. (2017). “Atypical forms” of benign epilepsy with centrotemporal spikes (BECTS): how to diagnose and guide these children. A practical/scientific approach. *Epilepsy Behav.* 75 165–169. 10.1016/j.yebeh.2017.08.001 28866336

[B47] Pavlou E. Gkampeta A. Evangeliou A. Athanasiadou-Piperopoulou F. (2012). Benign epilepsy with centro-temporal spikes (BECTS): relationship between unilateral or bilateral localization of interictal stereotyped focal spikes on EEG and the effectiveness of anti-epileptic medication. *Hippokratia* 16 221–224. 23935287 PMC3738727

[B48] Prehn-Kristensen A. Göder R. Fischer J. Wilhelm I. Seeck-Hirschner M. Aldenhoff J. (2011). Reduced sleep-associated consolidation of declarative memory in attention-deficit/hyperactivity disorder. *Sleep Med.* 12 672–679. 10.1016/j.sleep.2010.10.010 21697007

[B49] Rasch B. Born J. (2008). Reactivation and consolidation of memory during sleep. *Curr. Dir. Psychol. Sci.* 17 188–192. 10.1111/j.1467-8721.2008.00572.x

[B50] Rasch B. Born J. (2013). About sleep’s role in memory. *Physiol. Rev.* 93 681–766. 10.1152/physrev.00032.2012 23589831 PMC3768102

[B51] Ross E. E. Stoyell S. M. Kramer M. A. Berg A. T. Chu C. J. (2020). The natural history of seizures and neuropsychiatric symptoms in childhood epilepsy with centrotemporal spikes (CECTS). *Epilepsy Behav.* 103(Pt A) 1–9. 10.1016/j.yebeh.2019.07.038 31645314 PMC8087164

[B52] Scholle S. Zwacka G. Scholle H. C. (2007). Sleep spindle evolution from infancy to adolescence. *Clin. Neurophysiol.* 118 1525–1531. 10.1016/j.clinph.2007.03.007 17475551

[B53] Selvitelli M. F. Krishnamurthy K. B. Herzog A. G. Schomer D. L. Chang B. S. (2009). Sleep spindle alterations in patients with malformations of cortical development. *Brain. Dev.* 31 163–168. 10.1016/j.braindev.2008.06.006 18667284 PMC2722507

[B54] Shibagaki M. Kiyono S. Watanabe K. (1982). Spindle evolution in normal and mentally retarded children: a review. *Sleep* 5 47–57. 10.1093/sleep/5.1.47 7071451

[B55] Silvestri R. Gagliano A. Calarese T. Aricò I. Cedro C. Condurso R. (2007). Ictal and interictal EEG abnormalities in ADHD children recorded over night by video-polysomnography. *Epilepsy Res.* 75 130–137. 10.1016/j.eplepsyres.2007.05.007 17588723

[B56] Siripornpanich V. Visudtibhan A. Kotchabhakdi N. Chutabhakdikul N. (2019). Delayed cortical maturation at the centrotemporal brain regions in patients with benign childhood epilepsy with centrotemporal spikes (BCECTS). *Epilepsy Res.* 154 124–131. 10.1016/j.eplepsyres.2019.05.003 31129368

[B57] Smith A. B. Bajomo O. Pal D. K. (2016). Not necessarily benign: rolandic epilepsy. *Epilepsy Curr.* 16 254–255. 10.5698/1535-7511-16.4.254 27582666 PMC4988079

[B58] Spruyt K. (2021). Neurocognitive effects of sleep disruption in children and adolescents. *Child Adolesc. Psychiatr. Clin. N. Am.* 30 27–45. 10.1016/j.chc.2020.08.003 33223067

[B59] Staresina B. P. Bergmann T. O. Bonnefond M. Van Der Meij R. Jensen O. Deuker L. (2015). Hierarchical nesting of slow oscillations, spindles and ripples in the human hippocampus during sleep. *Nat. Neurosci.* 18 1679–1686. 10.1038/nn.4119 26389842 PMC4625581

[B60] Storz S. Wilhelm I. Critelli H. Feldmann M. Ramirez A. Ramantani G. (2020). Sleep-dependent memory consolidation in children with self-limited focal epilepsies. *Epilepsy Behav.* 113 1–7. 10.1016/j.yebeh.2020.107513 33129045

[B61] Strug L. J. Pal D. K. (2017). Reply to: is a microRNA-328 binding site in PAX6 associated with rolandic epilepsy? *Ann. Clin. Transl. Neurol.* 4 278–280. 10.1002/acn3.403 28382310 PMC5376750

[B62] Sud S. Sadaka Y. Massicotte C. Smith M. L. Bradbury L. Go C. (2014). Memory consolidation in children with epilepsy: does sleep matter? *Epilepsy Behav.* 31 176–180. 10.1016/j.yebeh.2013.12.012 24434309

[B63] Tassinari C. A. Cantalupo G. Rios-Pohl L. Giustina E. D. Rubboli G. (2009). Encephalopathy with status epilepticus during slow sleep: “the penelope syndrome”. *Epilepsia* 50(Suppl. 7) 4–8. 10.1111/j.1528-1167.2009.02209.x 19682041

[B64] Teixeira J. Santos M. E. (2018). Language skills in children with benign childhood epilepsy with centrotemporal spikes: a systematic review. *Epilepsy Behav.* 84 15–21. 10.1016/j.yebeh.2018.04.002 29730501

[B65] Tononi G. Cirelli C. (2006). Sleep function and synaptic homeostasis. *Sleep Med. Rev.* 10 49–62. 10.1016/j.smrv.2005.05.002 16376591

[B66] Tononi G. Cirelli C. (2014). Sleep and the price of plasticity: from synaptic and cellular homeostasis to memory consolidation and integration. *Neuron* 81 12–34. 10.1016/j.neuron.2013.12.025 24411729 PMC3921176

[B67] Urbain C. Di Vincenzo T. Peigneux P. Van Bogaert P. (2011). Is sleep-related consolidation impaired in focal idiopathic epilepsies of childhood? A pilot study. *Epilepsy Behav.* 22 380–384. 10.1016/j.yebeh.2011.07.023 21872533

[B68] Urbain C. Galer S. Van Bogaert P. Peigneux P. (2013). Pathophysiology of sleep-dependent memory consolidation processes in children. *Int. J. Psychophysiol.* 89 273–283. 10.1016/j.ijpsycho.2013.06.022 23810995

[B69] Varesio C. Zanaboni M. P. Salmin E. C. Totaro C. Totaro M. Ballante E. (2020). Childhood epilepsy with centrotemporal spikes: clinical and neuropsychological outcomes 5 Years after remission. *Diagnostics* 10:931. 10.3390/diagnostics10110931 33182826 PMC7696372

[B70] Verrotti A. Filippini M. Matricardi S. Agostinelli M. F. Gobbi G. (2014). Memory impairment and benign epilepsy with centrotemporal spike (BECTS): a growing suspicion. *Brain Cogn.* 84 123–131. 10.1016/j.bandc.2013.11.014 24362071

[B71] Wamsley E. J. Tucker M. A. Shinn A. K. Ono K. E. McKinley S. K. Ely A. V. (2012). Reduced sleep spindles and spindle coherence in schizophrenia: mechanisms of impaired memory consolidation? *Biol. Psychiatry* 71 154–161. 10.1016/j.biopsych.2011.08.008 21967958 PMC3561714

[B72] Weiner O. M. Dang-Vu T. T. (2016). Spindle oscillations in sleep disorders: a systematic review. *Neural Plast.* 2016:7328725. 10.1155/2016/7328725 27034850 PMC4806273

[B73] Wickens S. Bowden S. C. D’Souza W. (2017). Cognitive functioning in children with self-limited epilepsy with centrotemporal spikes: a systematic review and meta-analysis. *Epilepsia* 58 1673–1685. 10.1111/epi.13865 28801973

[B75] Wilhelm I. Diekelmann S. Born J. (2008). Sleep in children improves memory performance on declarative but not procedural tasks. *Learn. Mem.* 15 373–377. 10.1101/lm.803708 18441295

[B74] Zhao X. Chi Z. Chi L. Shang W. Liu X. (2007). Clinical and EEG characteristics of benign rolandic epilepsy in Chinese patients. *Brain Dev.* 29 13–18. 10.1016/j.braindev.2006.05.006 16806777

